# Social dynamics within decomposer communities lead to nitrogen retention and organic matter build-up in soils

**DOI:** 10.1038/ncomms9960

**Published:** 2015-12-01

**Authors:** Christina Kaiser, Oskar Franklin, Andreas Richter, Ulf Dieckmann

**Affiliations:** 1 Evolution and Ecology Program, International Institute for Applied Systems Analysis (IIASA), Schlossplatz 1, A-2361 Laxenburg, Austria; 2 Department of Microbiology and Ecosystem Science, University of Vienna, Althanstrasse 14, A-1090 Vienna, Austria; 3 Ecosystem Services and Management Program, International Institute for Applied Systems Analysis (IIASA), Schlossplatz 1, A-2361 Laxenburg, Austria; 4 Department of Forest Ecology and Management, Swedish University of Agricultural Sciences, SE-901 83 Umeå, Sweden

## Abstract

The chemical structure of organic matter has been shown to be only marginally important for its decomposability by microorganisms. The question of why organic matter does accumulate in the face of powerful microbial degraders is thus key for understanding terrestrial carbon and nitrogen cycling. Here we demonstrate, based on an individual-based microbial community model, that social dynamics among microbes producing extracellular enzymes (‘decomposers') and microbes exploiting the catalytic activities of others (‘cheaters') regulate organic matter turnover. We show that the presence of cheaters increases nitrogen retention and organic matter build-up by downregulating the ratio of extracellular enzymes to total microbial biomass, allowing nitrogen-rich microbial necromass to accumulate. Moreover, increasing catalytic efficiencies of enzymes are outbalanced by a strong negative feedback on enzyme producers, leading to less enzymes being produced at the community level. Our results thus reveal a possible control mechanism that may buffer soil CO_2_ emissions in a future climate.

## Abstract (web summary)

Microbial decomposers in soil provide the largest ecosystem flux of CO_2_ to the atmosphere, but interactions at the microscale are poorly understood. Here, the authors use a computer modelling approach to show that social interactions among microbes buffer changing environmental conditions.

## Graphical abstract

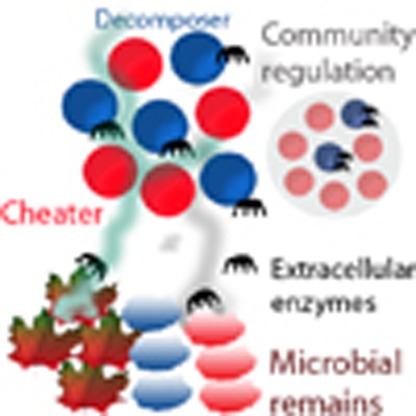

In biogeochemistry, microbial decomposition of organic matter has traditionally been modelled using first-order decay rates based on the chemical quality of the litter. Over the last decades, it has become increasingly evident that physiological processes and microbial responses to environmental conditions control decay rates, rather than litter chemistry^1^,^2^,^3^. Consequently, during the last decade conceptual models with a more explicit implementation of microbial controls, such as microbial biomass, extracellular enzyme activities and microbial physiology have been developed^4^,^5^,^6^,^7^,^8^,^9^,^10^,^11^,^12^,^13^ and the incorporation of microbial physiology into ecosystem models has repeatedly been suggested^4^,^5^,^14^.

First attempts to account for microbial physiology in large-scale biogeochemical models have demonstrated a strong impact on model predictions^5^,^6^,^11^,^15^. In particular, the scaling of microbial physiological parameters (regulating, for example, microbial growth efficiency or extracellular enzyme kinetics) with expected environmental change has led to largely diverging projections of future soil carbon (C) stocks^11^,^14^. The high sensitivity of model predictions to small changes in microbial physiological parameters highlights the need to better understand microbial mechanisms of organic matter decay in order to be able to make robust predictions of future soil C stocks.

The microbial physiology currently implemented in soil models is generally based on mechanistic concepts for single microbial cells^3^,^4^,^5^,^15^, which are scaled up to microbial communities. This follows the inherent assumption that the effects of physiological responses of microbes will be additive. Soil, however, is a complex system characterized by nonlinear interactions among functionally different microorganisms in a spatially structured and chemically heterogeneous environment. Albeit often neglected in microbial ecology, it is well known from other scientific disciplines such as physics, mathematics and theoretical biology that in complex systems nonlinear interactions between components at the micro-scale can lead to emergent system behaviour and new qualitative features at the macro-scale^16^,^17^,^18^. One key question for adding mechanistic details to soil models thus is: is it feasible simply to scale up physiological responses expected from single microbes to microbial communities? In a previous modelling study, we have shown that adaptations at the community level regulate the relative rates of C and nitrogen (N) recycling, which in turn improves nutritional conditions for microbes. The possibility of such self-regulating features of microbial communities is not yet considered in earth system models.

A specific characteristic of microbes is that they produce compounds that are released to their environment, for example, extracellular enzymes for the deconstruction of polymeric resources, polysaccharides for biofilm formation or quorum-sensing molecules^19^. Once released by the producing microorganism, these compounds become functionally available to other microbes in their surroundings^20^,^21^,^22^. The inevitable production of such ‘public goods' fosters social (synergistic and exploitive) interactions among microbes^19^. Experiments have shown that subpopulations of microbial ‘cheaters', which exploit public goods in which they did not invest resources, arise quickly whenever microbes producing these goods are present^23^,^24^,^25^. Microbial ‘cheaters', as a special form of opportunistic microbes, are thus an inevitable part of any microbial decomposer community. In a pioneering study, Allison demonstrated through individual-based modelling that competition between cheaters and microbes producing extracellular enzymes constrains the decomposition of complex compounds^13^. Taking this approach one step further, here we examine how social interactions at the micro-scale can affect and control large-scale fluxes and dynamics of C and N during organic matter turnover.

We use a recently developed individual based, spatial and stoichiometrically explicit model, which simulates C and N turnover during litter decomposition at the μm-scale in a spatially structured environment. In our model, ‘decomposers' produce extracellular enzymes to break down complex organic compounds, which are either of plant origin (primary substrate) or dead microbial cells (microbial remains, secondary substrate). The products of this enzymatic activity become available to nearby microbes via diffusion, allowing competitive and synergistic interactions at the micro-scale, which lead to emergent system dynamics at the macro-scale. We define ‘cheaters' as microbes investing less into extracellular enzyme production than decomposers, which means that they benefit from the investments of their competitors^26^. Our results demonstrate that the presence of microbial cheaters not only slows down decay rates, but also significantly increases the accumulation of N-rich microbial products during litter decay. Moreover, the presence of microbial cheaters made the decomposer system behave like a buffer: any increase in extracellular enzyme efficiency is outbalanced by increased abundances of cheating microbes, which lowers the amount of enzymes produced at the community level. Our results suggest that microbial cheaters may play an essential, but so far overlooked, role for C and N cycling in terrestrial ecosystems.

## Results

### Social interactions slow down decay rates

**Figure 1:**
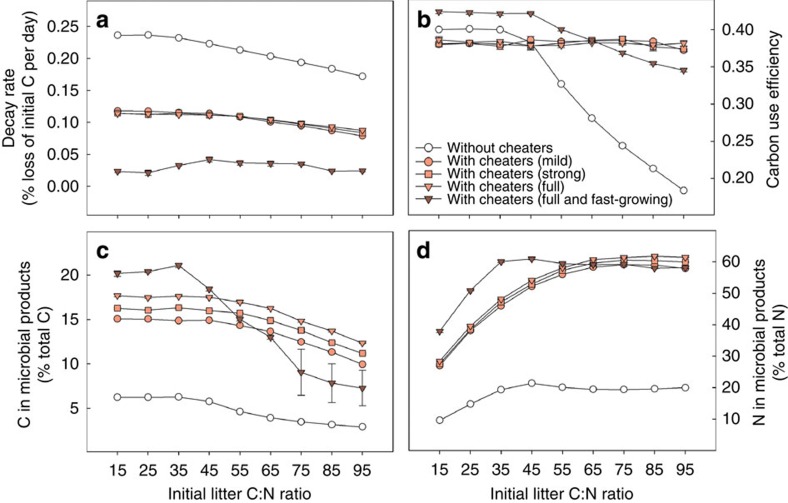
Social dynamics among microbial decomposers affect C and N turnover rates. Effect of microbial cheaters on (**a**) decay rates, (**b**) community carbon use efficiency (both time-averaged over model runs until 60% C loss), (**c**) C in microbial products and (**d**) N in microbial products (both aggregated over the grid at the point of 60% total C loss). Open circles: no cheaters, that is, all microbes have equal extracellular enzyme production rates of 0.12 (given as fraction of C uptake after deduction of maintenance respiration invested into extracellular enzyme production). Light-red symbols: mixed communities of enzyme producers with production rates of 0.12 and cheaters with enzyme production rates of 0.04 (‘mild', circles), 0.02 (‘strong', squares) or 0 (‘full', triangles), which otherwise have the same traits as enzyme producers. Dark-red triangles: mixed communities of enzyme producers and cheaters with enzyme production rates of 0 with a higher maximum growth rate compared with enzyme producers (Table 1). Error bars indicate model stochasticity by displaying s.d.'s among five independent model runs (error bars smaller than symbol size are not displayed).

The presence of cheating microbes in our model has a strong negative effect on overall litter decay rates across all initial litter C:N ratios (Fig. 1a). If cheaters possess the same functional traits as the main enzyme producers (except for enzyme production), the model predicts that decay rates are reduced by around 50%, no matter if the former are only partly or fully cheating (Fig. 1). If cheating microbes have a higher maximum growth rate than the main enzyme producers, as often observed for opportunistic microbes^27^, the slowing-down effect is magnified due to cheaters being more competitive, with decay rates being reduced by up to 90%. Initial litter N content also influences decay rates in the model: decay rates decrease with increasing initial litter C:N ratios, particularly in the absence of cheaters, due to increasing N limitation (Fig. 1a). The presence or absence of microbial cheaters, however, has a far stronger influence on decay rates than initial litter N content (Fig. 1a). Decay rates slow down specifically at low initial litter C:N ratios when fast-growing cheaters are present, as fast-growing microbes are especially competitive at high N availability^28^ (Fig. 1a, dark-red triangles). When initial C:N ratios are high, however, fast-growing cheaters are less competitive compared with cheaters that grow at the same (slow) rate as enzyme producers, because slow-growing microbes cope better with N limitation in our model^28^. The negative influence of fast-growing cheaters on decay rates is thus diminished when C:N ratios are high, which is visible from decreasing microbial products and carbon use efficiency (CUE), both coming closer to the levels seen in the absence of cheaters (Fig. 1c,d).

### Cheaters alter spatial dynamics

**Figure 2:**
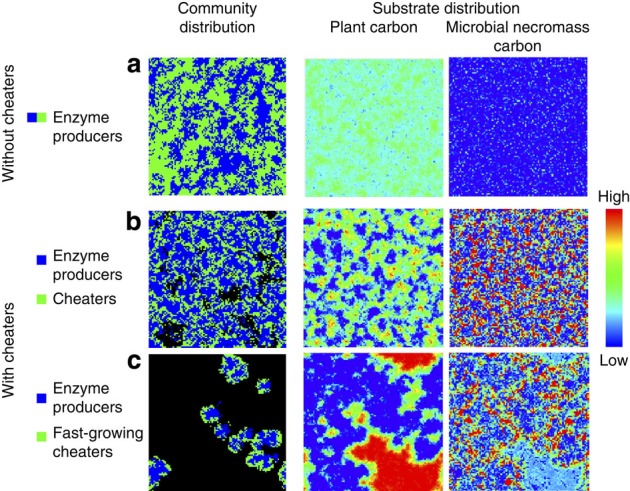
Spatial distributions of microbes and substrate in litter decomposition. Distributions with and without microbial cheaters. ‘Community distribution': each blue or green pixel depicts a model microsite occupied by microbes of a certain functional group, while black pixels depict empty microsites. ‘Substrate distribution': spatial distributions of the two complex substrates considered in the model (remaining plant material and microbial necromass); the colours of pixels indicate relative substrate concentrations according to the colour bar on the right. Snapshots of these distributions are shown for model runs in three different scenarios. (**a**) In the first scenario, without cheaters, all microbes produce extracellular enzymes at the same rate (here the microbes depicted in blue and green have identical functional traits; the community distribution is functionally homogeneous and the shown pattern just illustrates colony growth of initially randomly distributed microbes depicted in blue and green). (**b**) The second scenario considers a mixed community of enzyme producers (blue, with an enzyme production rate of 0.12) and cheaters (green, with an enzyme production rate of 0), only differing in their enzyme production rates. (**c**) In the third scenario, cheaters additionally have a faster maximum growth rate and a higher N demand compared with enzyme producers. All model runs start with microbes being randomly distributed across the grid. Each panel shows the model grid of 100 × 100 microsites at the time when 60% of the initial C is decomposed, that is, respired (**a**: 287 days, **b**: 556 days, **c**: 5,625 days). Consequently, the total amount of C stored across the two shown compound substrate pools, ‘plant carbon' and ‘microbial necromass carbon', is the same for all three scenarios at the time these snapshots are taken; only spatial distributions and relative allocations to these substrate pools differ. The presence of cheaters generally leads to a higher heterogeneity of substrate and to a higher relative allocation of C to the microbial necromass pool, which in turn leads to a higher proportion of N kept in the system (see main text). Fast-growing cheaters strongly constrain the dispersal of enzyme producer patches, leading to a different spatiotemporal dynamics of microbes and substrate.

Cheaters in our model preferentially occur along the edges of growing patches of main enzyme producers (Fig. 2, Supplementary Movie 1). This pattern occurs because edges of enzyme-producer patches represent zones of high availability of diffusive compounds, making them highly competitive areas for enzyme producers and cheaters alike. Competition for space along the edges, however, constrains the expansion of enzyme-producer patches (Fig. 2). This hindered growth of decomposer patches exacerbates the negative effect of cheaters on decomposition rates in our model (beyond pure resource competition), in particular when cheaters have higher maximum growth rates than decomposers (Figs 1a and 2). Over time, the presence of cheaters generally leads to a higher heterogeneity of both the remaining primary substrate and the accumulating microbial products (Fig. 2).

### Social interactions increase N and C accumulation

**Figure 3:**
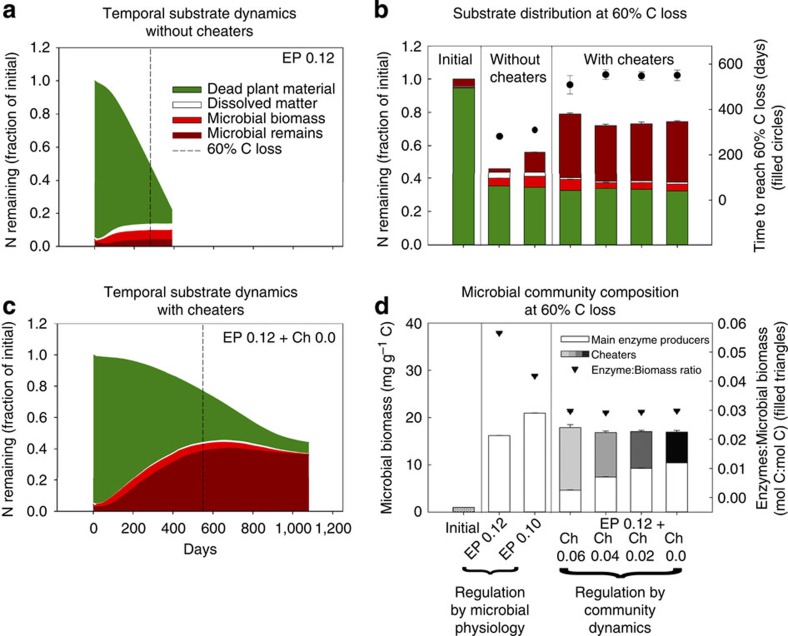
Nitrogen dynamics in model communities with and without microbial cheaters. (**a**,**c**) Temporal dynamics of N pools during litter decomposition. The two dashed lines indicate the times when 60% of C is decomposed. (**b**) Distributions of N in the remaining litter at 60% C loss in different model scenarios. (**d**) Microbial community compositions establishing in different model scenarios (shown abundances are time-averaged over model runs). The model is run either without cheaters, that is, with enzyme producers (EP) only (with enzyme production rates, given by the fraction of C uptake after deduction of maintenance respiration, of 0.12 or 0.10), or for mixed communities of enzyme producers and cheaters (Ch) initially consisting of 50% enzyme producers (with enzyme production rates of 0.12) and 50% cheaters (with enzyme production rates of 0.06, 0.04, 0.02 or 0). Filled circles: days until 60% of initial C is decomposed. Filled triangles: ratio of extracellular enzymes to microbial biomass (averaged over time). The initial litter C:N ratio equals 55. Error bars in **b** and **d** indicate model stochasticity by displaying s.d.'s among five independent model runs (error bars smaller than symbol size are not displayed).

The presence of microbial cheaters considerably improves C and N use efficiency of the microbial community, especially at low N availability. Without cheaters, community carbon use efficiency (CCUE) tends to decrease with increasing N limitation, that is, with increasing litter C:N ratio (Fig. 1b). This is due to the overflow mechanism implemented in our stoichiometric model at the microbial physiology level: N limitation leads to a greater proportion of C being respired, as it cannot be used for biomass build-up^4^,^29^. However, in the presence of cheaters, the emerging community dynamics increase the percentage of N-rich microbial remains and products in the remaining litter (Fig. 1c,d). This lowers the C:N ratio of dissolved matter, which is the result of enzymatic decomposition of complex substrates, that is, plant material with a relatively high C:N ratio and microbial remains with a relatively low C:N ratio. This, in turn, enables microbes to overcome N limitation, thereby allowing a more efficient use of C for biomass growth, which makes overflow respiration obsolete (Fig. 1b). As the accumulating microbial products and remains have a relatively low C:N ratio, N is retained to a greater extent than C, reflected in a higher C loss compared with N loss, when cheaters are present (Supplementary Fig. 1). Not only relative, but also absolute pool sizes of secondary substrates increase dramatically when cheaters are present: about five times more N accumulates in secondary microbial products when cheaters are present, thereby almost doubling the N content of the remaining litter compared with when cheaters are absent (Fig. 3). For example, at 60% C loss, around 80% of the initial N is kept in the system with social interactions (Fig. 3c), compared with only 45% without (Fig. 3a). With cheaters, it takes about twice as long to reach that stage of 60% C loss, and once attained, not only N, but also a greater part of the remaining C is stored in microbial remains (around 18%, as compared with 7% without cheaters, Fig. 1c,d).

### Underlying mechanism

**Figure 4:**
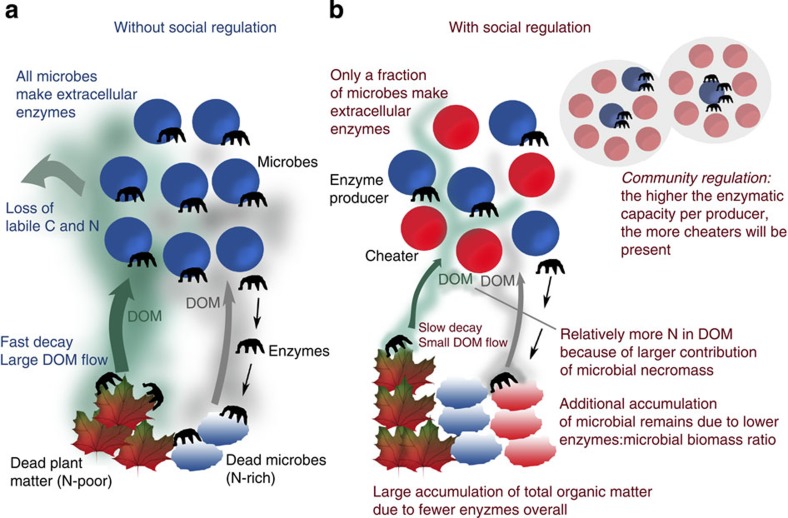
Effects of social regulation on microbial organic matter decomposition. (**a**) Without social regulation (all microbes produce enzymes), turnover rates are high due to a direct positive feedback of enzyme production on microbial growth. The accumulation of microbial remains is limited by a relatively high ratio of enzymes to microbial biomass. The consequently large DOM pool features losses of labile C and N. (**b**) When cheaters are present, only a (self-regulated) fraction of microbes produces enzymes, resulting not only in a lower total amount of enzymes, but also in a lower amount of enzymes per microbial biomass, leading to a lower amount of enzymes per dead microbial biomass (microbial remains). While the lower total amount of enzymes slows down decay rates, resulting in a smaller DOM pool and less loss over time of C and N by leaching, the lower ratio of enzymes to microbial remains increases the pool of N-rich microbial remains in relation to N-poor plant material, which in turn lowers DOM C:N ratio and alleviates microbial N limitation.

Closer investigation of C and N flows in our model reveals that the accumulation of microbial remains is closely linked to the ratio between microbial biomass and extracellular enzymes responsible for degrading dead microbial biomass. If that ratio is high, a small number of enzymes face a large amount of dead biomass, resulting in a slow degradation of the latter. Conversely, if that ratio is low, a high number of enzymes more quickly degrade a smaller amount of microbial remains (Fig. 4, Supplementary Fig. 2). In traditional decomposition models, which represent microbial biomass as one pool, as well as in our model without cheaters, the fraction of enzymes produced per total microbial biomass can be controlled by a parameter. Manipulating this parameter (*E*_fr_), that is, reducing the amount of C that microbes allocate to enzyme production, indeed increases the accumulation of microbial remains when cheaters are absent (Fig. 3, EP 0.12 and EP 0.10 at ‘Regulation by microbial physiology'). This shows the general sensitivity of the accumulation of microbial remains to the amount of enzymes produced per total microbial biomass, which inevitably affects the ratio of active enzymes to dead microbial biomass. The key result of our model analysis is, however, that in a mixed community of enzyme producers and cheaters community-level adaptations emerge: these alter the ratio between enzyme producers and cheaters in a way that downregulates to a minimum the total amount of enzymes produced per total microbial biomass (Fig. 3, ‘Regulation by community dynamics'), leading to the observed increased accumulation of microbial remains.

### Social dynamics buffer extracellular enzyme reaction rates

**Figure 5:**
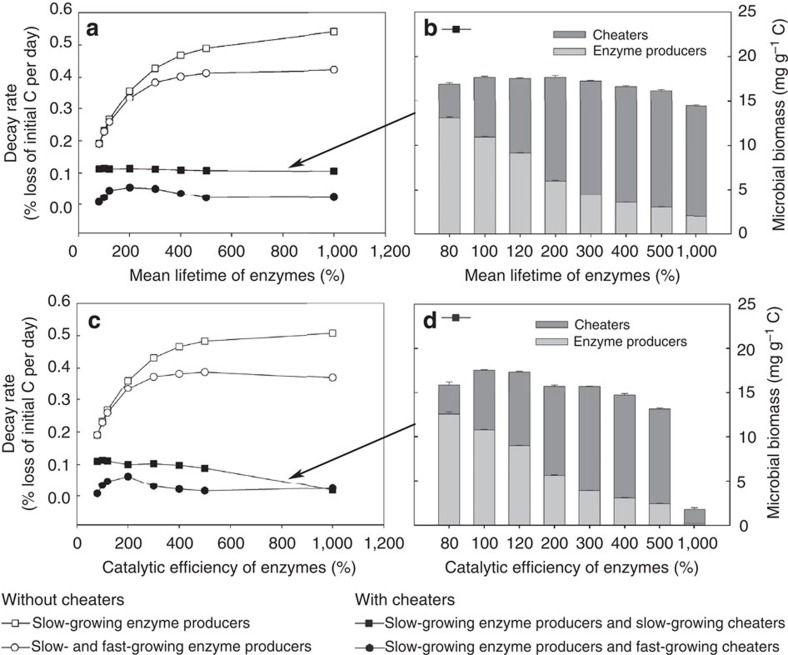
Microbial social dynamics buffer decay rates at varying levels of extracellular enzyme efficiency. (**a**,**c**) Decay rates derived from model scenarios with varying mean lifetimes of extracellular enzymes or varying levels of catalytic efficiency (100% mean lifetime=83 h for all enzymes; 100% catalytic efficiency=0.33, 0.63 and 0.3 mol substrate C per mol enzyme C per hour for enzymes that degrade plant material, C-rich microbial remains and N-rich microbial remains, respectively). Decay rates are measured in terms of % loss of initial C per day (averaged over time, until 60% C loss). Open symbols: without cheaters. Filled symbols: with cheaters. Open squares: population of enzyme producers with uniform growth rates. Open circles: mixed community of slow- and fast-growing enzyme producers. Filled squares: mixed community of enzyme producers (with an enzyme production rate of 0.12) and cheaters (with an enzyme production rate of 0). Filled circles: cheaters additionally have a faster maximum growth rate and a higher N demand compared with enzyme producers. For details see Table 1. (**b**,**d**) Microbial community compositions establishing in the presence of cheaters (which are functionally equivalent to enzyme producers except for their enzyme production rates), time-averaged over model runs. Error bars indicate model stochasticity by displaying s.d.'s among five independent model runs.

Enzyme kinetics are sensitive to temperature, with higher temperatures accelerating enzyme reaction rates^3^,^11^,^15^. However, it is not well understood how the observed intrinsic temperature sensitivity of enzyme kinetics translates into a measurably lower ‘apparent' temperature sensitivity of microbial decomposition of soil organic matter^3^,^15^. To evaluate the interaction of social community dynamics with changing enzyme reaction rates, we vary two model parameters that control the catalytic efficiency of enzymes: the catalytic rate constant *k*_cat_ of enzymes (the higher this parameter, the more units of substrate can be decomposed per enzyme and time, which is analogous to *V*_max_ divided by the amount of enzymes) and the mean lifetime 1/*k*_enz_ of enzymes (the higher this parameter, the more substrate can be decomposed per unit of enzyme). Without cheaters, decay rates in our model increase, as expected, with increasing enzyme reaction rates, until saturation is reached (Fig. 5). At the same time, CCUE decreases due to increased overflow respiration (Supplementary Fig. 3). With cheaters, no increase in decay rates with increasing enzyme reaction rates and no decrease in CCUE are observed in our model. Our results thus show that the presence of microbial cheaters effectively buffers even extreme increases in enzyme reaction rates (Fig. 5). This response is caused by a positive feedback of enzyme efficiency on cheaters abundance: the more substrate each unit of extracellular enzyme degrades, the greater is the proportion of cheaters that can be sustained in the microbial community. This in turn reduces the absolute amount of extracellular enzymes in the system. This community-inherent mechanism leads to the surprising outcome that the overall rate of enzyme activity at the community level is essentially independent of the biochemical efficiency of individual extracellular enzymes (Fig. 5).

## Discussion

Our results demonstrate that the microbial decomposer system has a high capacity for self-regulation. Social interactions between enzyme producers and cheaters create a powerful community feedback, which facilitates the build-up of C and N in N-rich microbial residues and buffers decomposition rates against variations in extracellular enzyme efficiencies. The interesting conclusion from this is that the ubiquitous presence of opportunistic, seemingly ‘useless' soil microbes, which exploit the decomposer system, may be key for some important ecosystem services, such as soil C sequestration, N retention and the acclimation of decomposition rates to changing environmental conditions.

Previous model-based studies have already shown that the presence of cheating microbes constrains enzyme-catalysed decomposition^13^,^28^,^30^. Our results reinforce and extend this finding by showing that social dynamics between enzyme-producing and cheating microbes can firmly downregulate the total amount of enzymes produced at the community level, which, as a consequence, maximizes the accumulation of microbial remains during the decomposition of plant litter. Our model differs from other individual-based microbial enzyme models that also addressed the question of social dynamics between microbial producers of extracellular enzymes and cheaters^13^,^30^ in two important ways.

First, our model includes complete recycling of C and N from dead microbial biomass and microbial products such as extracellular enzymes. In the first, seminal, modeling study targeting social dynamics within microbial decomposer communities dead microbial biomass and extracellular enzymes were removed from the community without being re-metabolized by new microbes^13^. Microbial processes in this model were thus mainly responding to external C and N input, which was provided at a constant rate. In our model, by contrast, an initial pool of dead plant material is degraded over time by microbial activity. Part of the primary substrate is incorporated into microbial biomass and transformed into microbial remains on cell death, which in turn serve themselves as substrate for microbes (Fig. 3, ref. 28). Consequently, microbial processes in our model are strongly regulated by internal recycling of C and N.

The second important difference is that complex substrates in our model contain both C and N (at a certain ratio), whereas complex substrates in previous individual-based microbial enzyme models^13^,^30^ consisted of either C or N (or phosphorus, P), which are degraded by C-, N- or P-specific extracellular enzymes independently from the other elements, the turnovers of C, N and P thus being decoupled. In our model, C and N fluxes are linked to each other due to explicit substrate stoichiometry: C and N are liberated by enzymatic activity in the same ratio in which they are present in the complex substrate. The overall C:N ratio of dissolved organic matter (DOM)—which is what microbes face—is thus a result of the ratio at which the different complex substrates (that is, plant material with a relatively high C:N ratio and microbial remains with a relatively low C:N ratio) are degraded. This ratio depends both on the relative availability of the different complex substrates and on the relative availability of the substrate-specific enzymes. While the latter can be influenced by dynamics of different functional groups producing specific extracellular enzymes^28^, the former is, as we show in this study, strongly influenced by the presence of cheaters, which increase the ratio of microbial remains to the remaining plant material. Both aspects, recycling of microbial remains and stoichiometric constraints imposed by linking C and N in complex substrates, enable a strong community-level feedback on microbial decomposition processes (and vice versa) in our model.

Notably, the ratio of extracellular enzymes to microbial biomass in our model settles at a similar minimum value across all our scenarios that include cheaters—independent of the degree of their cheating. As total microbial biomass stays about the same with or without cheaters, collective enzyme production seems to be downregulated to a point where the same total amount of biomass could still be sustained, but waste is minimized. This finding is consistent with the ‘black queen hypothesis'^31^, which suggests that evolution favours losses of metabolic pathways for producing public goods, until a community's production of the public good is just sufficient to support it at equilibrium. Although the overall decomposition process takes considerably longer with cheaters than without, resources are used more efficiently, so that in total a greater number of microbes can thrive on it.

At the cellular level, there is thought to be a metabolic trade-off between the rate of resource acquisition and the net yield of energy: higher rates are often coupled to lower yields and vice versa^32^,^33^. High-rate/low-yield organisms are understood to have an advantage over low-rate/high-yield organisms when competing for external resources^32^, leading to communities with overall inefficient resource use—an evolutionary dilemma well known as the ‘tragedy of the commons'^34^. While the rate-yield trade-off can be explained by thermodynamic principles at the cellular level^32^, our model shows a similar trade-off at the community level: high rates of enzyme production and microbial turnover are coupled with low efficiency of resource use (that is, low net yield of energy) in communities without cheaters, whereas low rates of enzyme production and microbial turnover are coupled with high efficiency of resource use in communities with cheaters. Lower rates are linked to higher efficiencies in our model because the cycle of matter is less leaky at slower turnover rates, with more C and N being stored in complex compounds, rather than in labile compounds (where they are prone to losses), at any one time (Supplementary Fig. 2). The interesting additional insights our findings offer relative to earlier studies is that cheaters pull the whole decomposer system to the efficient side of the trade-off, such that, unlike in previous analyses, the selfish interest of individuals (cheaters) in a public good does not lead to a tragedy of the commons, but to its opposite—a more efficient resource use for all.

Explicit space is known to facilitate the existence of cooperators or producers in the face of cheaters^13^,^26^,^35^ and was also a prerequisite for the stable coexistence of producers and cheaters in our model: increasing diffusion rates, as well as a well-mixed system, lead to a dominance of cheaters, which crash the community (not shown) similar to previous results^13^,^35^. In our model, cheaters predominately grow at the edge of decomposer patches, which are hotspots of competition for resources and space (Fig. 2, Supplementary Movie 1). Similar spatial dynamics between cheaters and producers of public goods have been observed not only in model-based studies, but also in empirical studies^13^,^30^,^36^. In general, our model's behaviour is consistent with recent empirical work demonstrating that enzyme-producing yeast cells not only exhibit stable coexistence with invading cheaters, but also that a relatively small fraction of enzyme producers (10%) supports a majority of cheaters at the eco-evolutionary steady state^37^,^38^.

A central finding of our study is that the ratio of extracellular enzymes to microbial biomass may be a key control of the amount of microbial necromass accumulating during the decomposition of plant litter. During the last decades, both conceptual and empirical research has sought to identify possible microbial mechanisms influencing C and N sequestration in soils. Microbial substrate use efficiency or CUE, with the latter measuring the fraction of C uptake invested into microbial biomass build-up, has been recognized as a potential driver of the capacity of soil to sequester C in the long run^5^,^39^,^40^,^41^,^42^. Recently, microbial turnover rates have additionally been implicated in soil C sequestration^42^. Here, we propose to extend these concepts by the ratio of extracellular enzymes to microbial biomass. This ratio, which appears to be dynamically regulated when microbial cheaters are present, controls, as we have shown here, the degradation rate, and thus the possible accumulation, of microbial remains. The fate of microbial remains, in turn, is key for C and N cycling in soil^43^,^44^,^45^, because microbial residues are thought to be the main precursor of stable soil organic matter formation^2^,^39^,^43^,^44^. Social microbial dynamics between enzyme producers and cheaters may thus be important for the long-term accumulation and storage of soil organic matter. In particular, the trapping of N in complex microbial remains prevents its loss to the environment—by leaching of N from DOM or dissolved inorganic nitrogen (DIN)—thereby increasing N retention in the system. Interestingly, in our model the downregulation of the ratio of extracellular enzymes to microbial biomass by social dynamics feeds back on CCUE and stabilizes it at a relatively high level.

We evaluated the self-regulation capacity of the decomposer system with cheaters by varying the catalytic efficiency (*k*_cat_) and longevity (1/*k*_enz_) of extracellular enzymes. Previous individual-based microbial enzyme models have revealed that higher enzyme production rates of decomposers increase cheater abundance and in turn lower decay rates^10^,^13^,^30^. This is because increased enzyme production is associated with increased costs for producers, which lower their competitive ability. Here we went one step further and specifically tested the effect of increased degradation ‘power' of individual enzymes, but at unchanged costs for enzyme producers. This analysis is based on the rationale that enzyme kinetics are sensitive to temperature change^3^,^15^ and that kinetic changes are therefore sometimes incorporated into microbial enzyme models (at the bulk level) to predict responses to climatic changes^5^,^14^.

Our results show that increased enzyme efficiencies are effectively outbalanced by a community response, leading to a reduction of total enzyme production at the community level. This feedback is not necessarily driven by lower competitive capabilities of producers due to increased enzyme costs. Rather, cheaters always benefit from increased enzyme activities, because it makes more resources available to them. From an evolutionary perspective, this raises the question whether the co-evolution with cheating microbes could have prevented evolution towards more efficient soil enzymes, as more efficient enzymes would not increase the fitness of their producers.

The self-regulating social dynamics in our model cause overall decay rates to become completely independent of catalytic enzyme strengths (Fig. 5). This indicates that neither the catalytic power of individual enzymes nor their mean lifetime may be as important for decay rates as often thought. For instance, a possible positive Arrhenius effect of temperature on enzymatic reaction rates in soils under global warming (that is, increasing catalytic efficiencies of enzymes with warming) could be outweighed by social dynamics within decomposer communities, and thus turn out to be less significant than previously thought^3^. This finding is consistent with the well-known observation that the apparent temperature sensitivity of decomposition is lower than the intrinsic temperature sensitivity expected from enzyme kinetics and substrate chemistry^3^,^15^. The measured thermal adjustments of the microorganisms may thus not only reflect shifts in community composition towards taxa adapted to warmer temperatures, as has been proposed recently^15^, but may also include the feedback of cheaters counteracting temperature-induced increases in enzyme efficiencies.

We conclude that to understand and predict organic matter decay it is necessary to go beyond microbial physiology and to implement community-level regulation in biogeochemical models^5^,^11^,^14^,^46^. Without accounting for such regulation, both extracellular enzyme kinetics and microbial CUE exert a strong control on decay rates in most biogeochemical models, making model predictions highly sensitive to variations in these parameters^5^,^6^,^11^,^14^. Our work shows, by contrast, that extracellular enzyme kinetics and microbial CUE may both be subject to regulation at the community level, which can overrule physiological responses from individual microbes or changes in biochemical efficiencies of individual enzymes. Not accounting for community-level regulations may thus lead to a vast over- or underestimation of future C stocks.

Individual-based micro-scale models, as the one we have used in this study, enable valuable insights into possible interactions between microbial community dynamics and processes. Novel techniques facilitating single-cell analysis of microbes within complex communities are now becoming available; these allow quantifying microbial processes at the scales at which they occur^47^,^48^,^49^. Applying these methods to well-designed experiments targeting natural and manipulated decomposer communities will allow to test the hypotheses generated by this study and to advance our understanding of the controls of microbial decomposition of organic matter.

## Methods

### Individual-based and spatially explicit model

We use a recently developed individual-based, spatially and stoichiometrically explicit micro-scale model, calibrated with experimental data from a litter decomposition experiment^28^. The model is implemented as a process-based and object-oriented computer program in Java, which simulates C and N turnover during litter decomposition based on micro-scale interactions among individual microbes. Macro-scale C and N turnover rates and pool dynamics emerge from interactions among individual microbes at the micro-scale, rather than being calculated by stock and flow rate equations at the bulk soil level. A detailed description of model equations and algorithms can be found in the Supplementary Material (Supplementary Tables 1 and 2 and Supplementary Methods).

Briefly, the model simulates a 1 mm^2^ piece of decomposing organic matter as a grid of 100 × 100 ‘microsites', each measuring 10 μm × 10 μm × 10 μm and containing different types of complex organic compounds, bioavailable labile compounds, microbes and extracellular enzymes.

### Complex compounds

Complex organic compounds are either original plant material (primary substrate) or dead microbial cells and products (secondary substrate). While the former becomes depleted over a model run (as there is no new input of resources), the latter can accumulate over time. Two types of secondary substrate are considered in the model: C-rich microbial remains (containing cell walls, lipids, carbohydrates and others, with an overall C:N ratio of 150) and N-rich microbial remains and products (containing proteins, DNA, and RNA from dead microbial biomass, as well as denatured extracellular enzymes, with an overall C:N ratio of 5). The C:N ratio of the primary substrate (plant material) depends on the considered model scenario (initial litter C:N ratio).

### Extracellular enzyme activity

In each microsite, complex compounds are degraded by extracellular enzymes residing in the microsite, following Michaelis–Menten kinetics^5^,^50^,


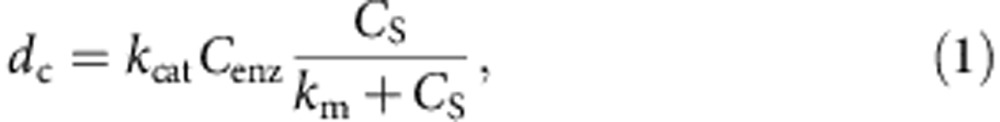


where *d*_c_ is the amount in the microsite of C released by the enzyme-catalysed breakdown of complex compounds per time step, *k*_cat_ (catalytic constant) is the number of enzymatic reactions catalysed per time step per enzyme (mol of substrate-C decomposed per mol of enzyme-C), *C*_S_ and *C*_enz_ are the amounts in the microsite of complex substrate and extracellular enzymes, respectively, and *k*_m_ is the half-saturation constant for the enzymes on the substrate. Amounts of C and N liberated by extracellular enzyme activity are added to the bioavailable DOM pool of the microsite. Extracellular enzymes are assumed to become inactive after some time, which is controlled by a first-order rate constant (*k*_enz_), whose inverse (1/*k*_enz_) therefore measures the mean lifetime of extracellular enzymes in the model.

### C and N uptake and processing by microbes

Microbes take up labile products of enzymatic breakdown (DOM), as well as DIN, subject to cell-size-specific maximum uptake rates and local availability (Supplementary Methods). From the resources taken up, maintenance respiration. calculated as a constant fraction of biomass C, is met first. A functional-group-specific fraction (*E*_fr_) of the remaining C and N uptake is invested into extracellular enzyme production, and any C and N remainder after that is invested into growth. Consequently, the smaller *E*_fr_, the more C and N resources can be used for growth.

### Waste metabolism

Stoichiometric imbalance between C:N in microbial uptake and microbial demand for respiration, enzyme production and growth results in either local N mineralization (excess N is released into the DIN pool of the microsite) or overflow respiration (excess C is respired)^4^.

### Diffusion

In every time step, 8/9 of the DOM and DIN in every microsite diffuses to its eight neighbouring microsites (each of which thus receives 1/9). The time step's length (3 h) was chosen so that the emerging diffusion coefficients match empirical diffusion coefficients^54^. A fraction of all diffusing elements is lost by leaching. For details on the diffusion algorithm, see Supplementary Methods of this study and in ref. 54.

### Microbial dispersal and turnover

Microbial cells that grow beyond a certain level divide and colonize neighbouring microsites. Microbes die either by starving (if their biomass falls below a minimum level) or by catastrophic death, regulated by a stochastic mortality rate. On cell death, the biomass C and N of the microbe is distributed among different substrate pools (C- and N-rich microbial remains, respectively, and DOM) within the microsite according to their functional-group-dependent biomass composition (Table 1).

### Initial values and model inputs

Complex substrates are initially homogenously distributed across the grid. Initial litter consists of 98.5% of plant material and 1.5% of microbial remains (Supplementary Table 1). There is no new input of organic material during a model run. The model stops when the amount of available substrate has become too low or too fragmented to support microbial activity, which usually occurs around 80% C loss (Supplementary Movie 1).

### Community carbon use efficiency

In every time step, CCUE is calculated from bulk C fluxes aggregated across the grid^29^,


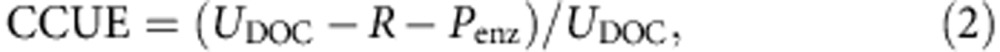


where *U*_DOC_ is the total amount of dissolved organic carbon taken up by all microbes on the grid, *R* is the total amount of C respired, and *P*_enz_ is the total amount of C released as extracellular enzymes. CCUE thus aggregates the CUEs of all individual microbes on the grid based on the sum of their C fluxes. In turn, the CUE of individual microbes are the result of the various microbial processes occurring in a model time step (Supplementary Methods), and therefore depends on the microbe's local situation and a few basic parameters. Specifically, the CUE of an individual microbe is a function of maintenance respiration (R_maint_, as a fraction of biomass), respiration-associated growth and enzyme production (R_ge_, as a fraction of C used for growth and enzyme production), and most importantly, local availability of C and N. In particular, a high C:N ratio of DOM (implying N limitation) leads to C overflow respiration, as excess C needs to be taken up and respired to gain N for growth, so CUE decreases. C limitation also decreases CUE: if not enough C is available for maximum growth, a relatively greater fraction of C is needed for maintenance respiration, which microbes need to secure before investing into new biomass growth.

### Model scenarios

We compare scenarios in which all microbes produce extracellular enzymes at the same rate (without cheaters, so no social dynamics are possible) with scenarios featuring mixed communities of initially 50% enzyme-producing microbes and 50% cheaters (thus allowing social dynamics). All communities are initially randomly distributed across the grid with a total of 16.7% of microsites being occupied. We define cheaters as microbes investing less into extracellular enzyme production than their competitors. We consider different levels of cheating, with cheaters producing enzymes at 1/2, 1/3 or 1/6 of the rate of main enzyme producers, or not producing any enzymes at all (full cheaters). Model outputs, such as decay rates and various pool sizes, are aggregated over the whole grid, describing the macro-scale behaviour of the system^28^.

Besides differences in enzyme production rates, two functional types of microbes are used in the model: fast growers and slow growers. Fast growers are assumed to have smaller cell sizes (resulting in higher turnover rates) and a lower C:N ratio (resulting in a higher N demand). Conversely, slow growers have larger cell sizes and a higher C:N ratio (Table 1). Most of our model scenarios are run with enzyme producers and cheaters being both slow growers, that is, they do not differ from each other in any trait other than their rate of enzyme production (Fig. 1 and Supplementary Fig. 1, except dark-red triangles, Figs 3 and 5, squares, Supplementary Fig. 3). To account for the fact that cheaters are likely to have faster growth rates than enzyme producers, we additionally include model scenarios with slow-growing enzyme producers and fast-growing cheaters (dark-red triangles in Fig.1 and Supplementary Fig. 1, filled circles in Fig. 5) or with slow- and fast-growing enzyme producers (open circles in Fig. 5).

## Additional information

**How to cite this article:** Kaiser, C. *et al.* Social dynamics within decomposer communities lead to nitrogen retention and organic matter build-up in soils. *Nat. Commun.* 6:8960 doi: 10.1038/ncomms9960 (2015).

## Supplementary Material

Supplementary Information Supplementary Figures 1-3, Supplementary Tables 1-2, Supplementary Methods and Supplementary References.

Supplementary Movie 1 Spatio-temporal dynamics of microbial decomposers and cheaters during a model run - Visualisation of spatio-temporal dynamics of microbial decomposers, cheaters and organic matter during a model run (simulating a 1 mm2 area of decomposing leaf litter). All panels represent the same grid of 100 x 100 microsites (each microsite is 10x10x10 μm). The large panel shows the distribution of two functional groups of soil microbes: blue, microbial enzyme producers; green, cheaters, which do not contribute to enzyme production. The two small panels depict substrate dynamics, which are closely linked to microbial community dynamics. Upper left: Distribution of initial plant C (Primary substrate), which becomes depleted over time; Lower left: Accumulation and turnover of microbial necromass C (Microbial remains). Parameter settings are as in Table 1, with cheaters being fast-growers.

## Figures and Tables

**Table 1:** Microbial functional traits used in model analysis.

**Parameter**	**Description**	**Fast growers**	**Slow growers**	**Unit**
Microbial cell composition and stoichiometry*				
*F*_DOM_	Fraction of cell biomass accounting for cell solubles (immediately available for uptake by other microbes on cell death, C:N=15)	0.06	0.06	
*F*_CC_	Fraction of cell biomass accounting for C-rich complex compounds, that is, cell wall compounds, lipids, starch (C:N=150)	0.52	0.37	
*F*_NC_	Fraction of cell biomass accounting for N-rich complex compounds, that is, proteins, DNA, RNA (C:N=5)	0.42	0.57	
*M*_CN_	Resulting biomass C:N	9.03	12.22	
				
*Microbial cell size and turnover rates*†				
*S*_max_	Size at which a microbial cell divides and colonizes a neighbouring microsite	10	100	fmol C
*S*_min_	Size at which a microbial cell dies from starving	1	10	fmol C
*c*	Maximum number of microbes in one microsite	3	1	
				
*Microbial enzyme production*				
*E*_fr_	Fraction of C uptake (after deduction of maintenance respiration) that is invested into enzyme production			
	Enzyme producers	0.12	0.12	
	Cheaters	0, 0.02, 0.04 or 0.06	0, 0.02, 0.04 or 0.06	
*E*_fPS_:*E*_fCR_:*E*_fNR_	Ratio in which specific enzymes are produced for the degradation of plant material : C-rich microbial remains : N-rich microbial remains			
	Enzyme producers	0.7:0.15:0.15	0.7:0.15:0.15	
	Cheaters (when also producing enzymes)	0.7:0.15:0.15	0.7:0.15:0.15	

^*^Chemical composition of prokaryotic and eukaryotic (for example, yeast) cells are based on ref. 27.

^†^Microbial cell sizes are based on refs 51, 52, 53. Microbial turnover rates are indirectly linked to cell size, as maximum C and N uptake rates are linked to the cell surface-to-volume ratio (which is higher in smaller cells). Maximum uptake rates are thus dynamic (depending on actual cell size). In addition, microbial mortality rates are inversely linked to maximum cell size, assuming that larger species invest more into defensive structures. Resultant uptake rates depend on maximum uptake rates and local C and N availability. For more details and all other model parameters, see Supplementary Methods.
