# Supplementary material for: Social dynamics within decomposer communities lead to nitrogen retention and organic matter build-up in soils
Source: Nat Commun. 2015 Dec 1;6:8960. doi: 10.1038/ncomms9960 (PMC4697322; doi:10.1038/ncomms9960)
Supplement: Supplementary Information — Supplementary Figures 1-3, Supplementary Tables 1-2, Supplementary Methods and Supplementary References. [file ncomms9960-s1.pdf]

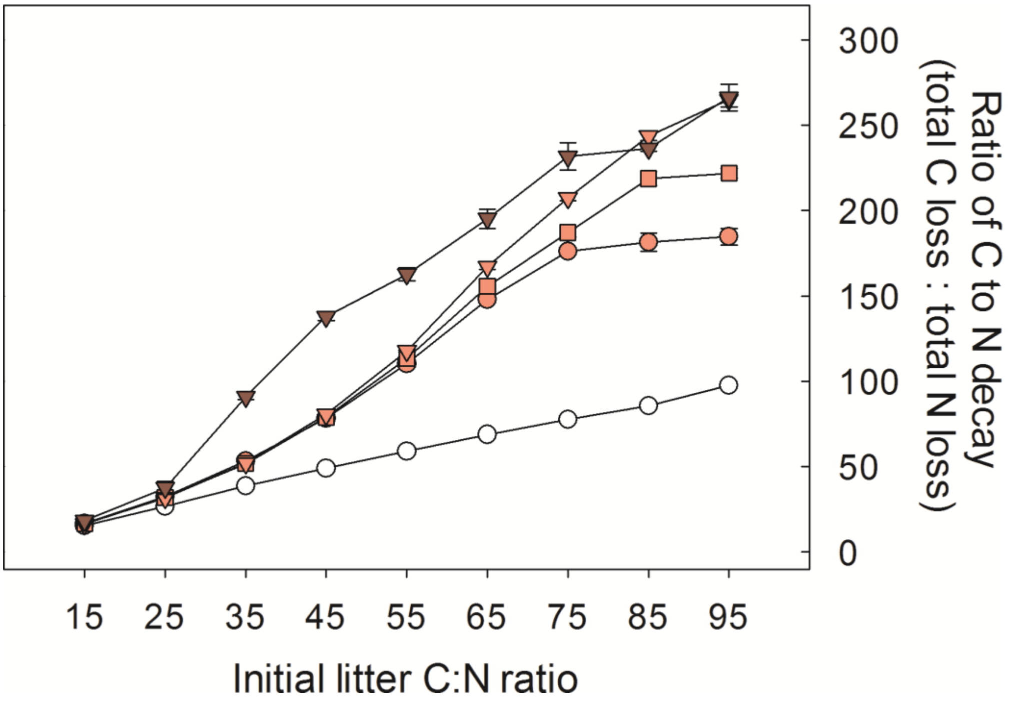

Supplementary Figure 1: The effect of microbial social dynamics on the ratio of carbon to nitrogen decay. The ratio of C to N decay is represented as the molar ratio of C and N loss during a model run (C and N are lost via respiration and leaching). With cheaters, Less N is lost compared to C than without cheaters. Open circles: No cheaters - all microbes have equal extracellular enzyme production rates (0.12, given as fraction of C uptake after deduction of maintenance respiration invested in extracellular enzyme production). Light red symbols: mixed populations of enzyme producers (production rate 0.12) and cheaters with enzyme production rates of 0.04 (circles), 0.02 (squares), or 0.0 (triangels) and otherwise the same traits as enzyme producers. Dark red triangles: mixed population of enzyme producers and cheaters (enz. prod. rates 0.0) with a faster maximum growth rate compared to enzyme producers (Table 1). Error bars display standard deviation of five model runs to account for model stochasticity (smaller than symbol size if not displayed).

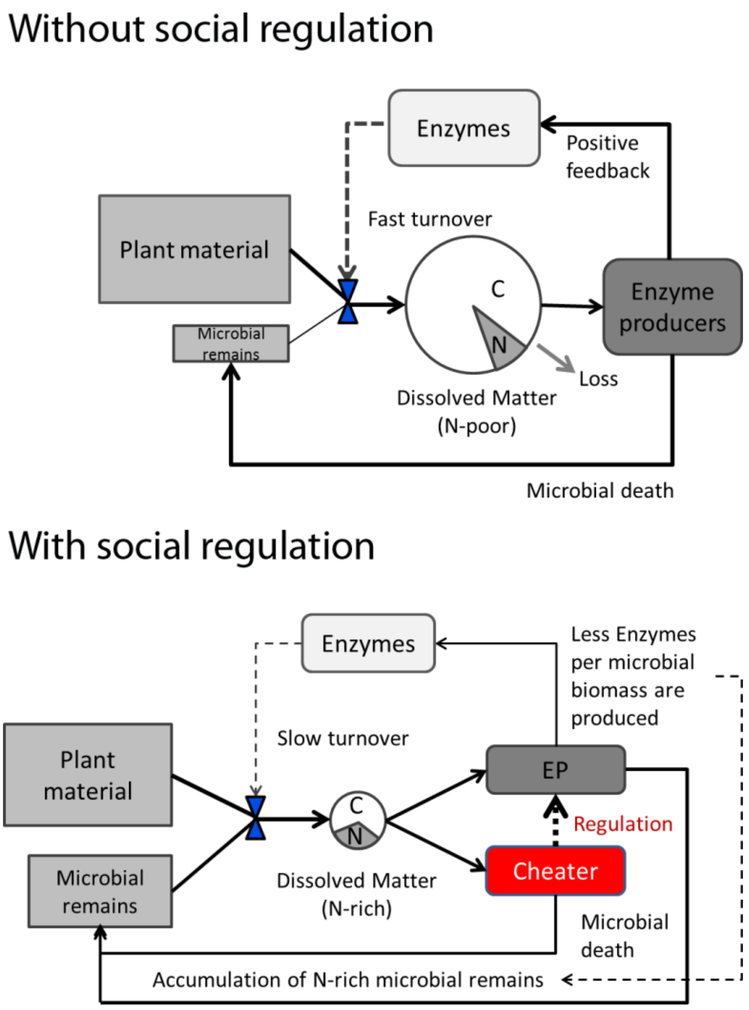

Supplementary Figure 2: Regulation of decomposition by social dynamics as a stock- and flow conceptual view. Without social regulation, i.e. without cheaters present, the production of extracellular enzymes by all microbes lead to a positive feedback and thus to a self-reinforcement of decomposition rates. As a consequence, initial plant material turns over quickly, and the build-up of a significant pool of microbial remains is prevented. Fast decomposition of complex organic matter create a relatively large pool of dissolved matter at any time point from which C and N is easily lost by leaching. By contrast, with social regulation, cheaters compete with enzyme producers thereby decreasing numbers of enzyme producers. While the total amount of microbial biomass is similar to the scenario without cheaters, the ratio of extracellular enzymes per microbial biomass is lowered. Lower absolute numbers of extracellular enzymes generally slow down organic matter turnover. The reduced ratio of extracellular enzymes to dead microbial cells particularly promotes the buildup of a N-rich microbial remains pool. As a result, nitrogen is trapped for longer times in complex organic matter pools, which increases N retention of the system. By altering the relative sizes of the (relatively N poor) plant material and the (relatively N rich) microbial-remains pool, the bioavailable DOM pool becomes more enriched with N, which in turn increases microbial carbon use efficiency. Overall, the regulating presence of microbial cheaters establishes a more efficient use and recycling of resources which allows a greater amount of microbial biomass to thrive over the time course of litter decomposition.

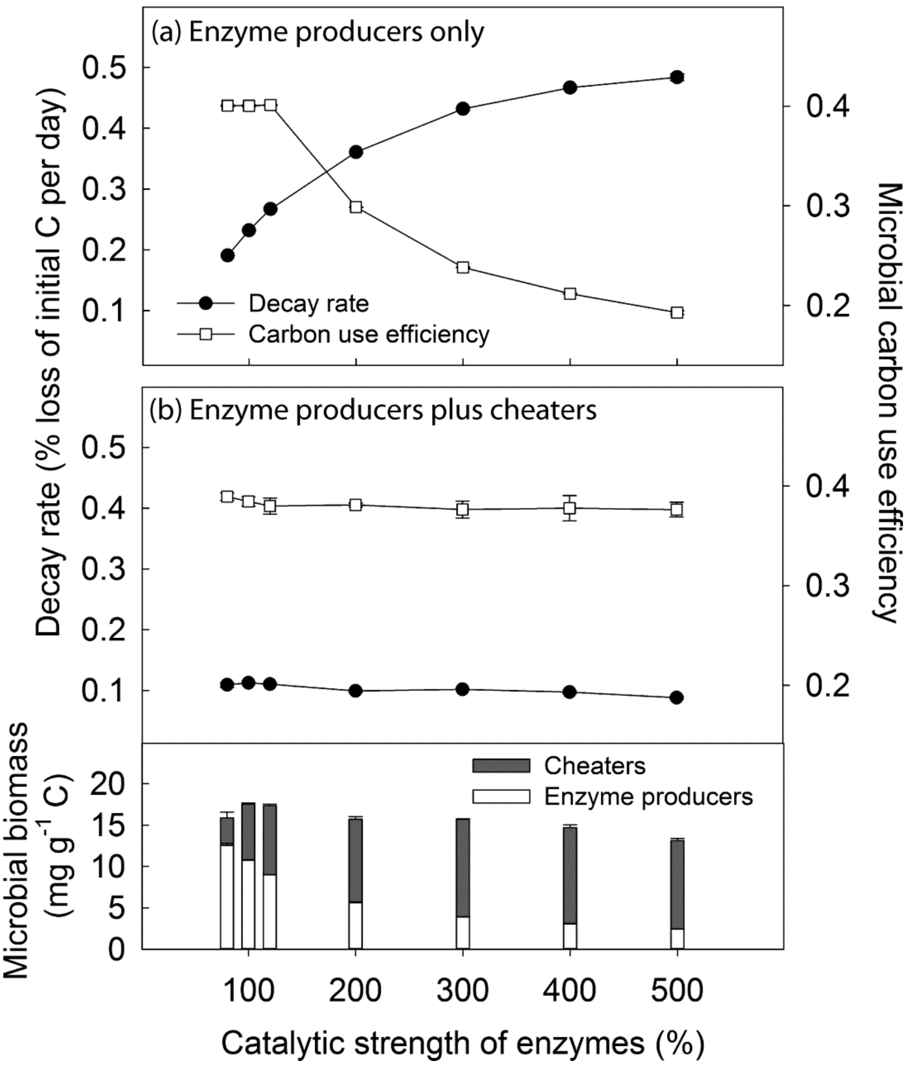

Supplementary Figure 3: Effect of microbial social dynamics on community carbon use efficiency during of litter decay. Aggregated results of model runs (until 60% C loss) with increasing levels of catalytic strength of extracellular enzymes with and without cheaters present (100% indicate reference values based on literature and previous model calibration [1], i.e. catalytic rate constant = 0.33, 0.63 and 0.3 mol substrate C mol enzyme C^{-1} $hour^{-1}$ for enzymes that degrade plant material, C- and N-rich microbial remains, respectively). Decay rates were calculated as % loss of initial C per day (averaged over time). Community carbon use efficiency (CCUE) was calculated each time-step as fraction of total microbial C uptake on the grid used for total microbial growth. Displayed is the average CCUE over the model's run time. Enzyme producers and cheaters only differed in their capacity to produce extracellular enzymes (Enzyme producers: 12% of C uptake after deduction of maintenance respiration, Cheaters: 0). Lowermost panel: Average community composition over time in the plus-cheaters scenario. Error bars display standard deviation of five model runs (smaller than symbol size if not displayed).

Supplementary Table 1: General model parameters.

Parameters	Description	Value	Unit
**Enzyme kinetics***			
k_{cat}: Number of enzymatic reactions catalyzed per enzyme (mol substrate-C decomposed per mol enzyme-C)			
k_{cat_PS}	k_{cat} of enzymes degrading primary substrate (Plant)	0.66	$timestep^{-1}$
k_{cat_CMR}	k_{cat} of enzymes degrading C-rich microbial remains	1.89	$timestep^{-1}$
k_{cat_NMR}	k_{cat} of enzymes degrading N-rich microbial remains	0.9	$timestep^{-1}$
k_m: Half-saturation constant for substrate in one microsite			
k_{m_PS}	k_m Primary substrate (Plant)	0.29	fmol C
k_{m_CMR}	k_m C-rich microbial remains	0.28	fmol C
k_{m_NMR}	k_m N-rich microbial remains	0.25	fmol C
k_{enz}	First order rate constant for inactivation of enzymes	0.036	$timestep^{-1}$
**Microbial physiology**			
R_{maint}	Maintenance respiration (fraction of biomass)	0.008	$timestep^{-1}$
R_{ge}	Respiration for growth and enzyme production (fraction of carbon used for growth/enzyme prod.)	0.26	$timestep^{-1}$
U_{max}	Basic maximum uptake rate (as fraction of biomass, to be multiplied with individual surface:volume ratio)	0.0057	$timestep^{-1}$
m_f[¶]	Relation between mortality rate and the inverse of maximum biomass per MS	0.34	
**Leaching**			
frL	Fraction of diffusing DOM that is lost by leaching	0.0088	$timestep^{-1}$
**Initial pool sizes in each microsite**		Initial amounts	
C_{Enz}	Active extracellular enzymes	0.5	fmol C
C_{CMR}	C-rich microbial remains	100	fmol C
C_{NMR}	N-rich microbial remains	30	fmol C
C_{DOM}	Bioavailable dissolved organic matter (initial C:N ratio = 8)	7	fmol C
C_{PS}	Primary substrate (Plant material) C	8333[§]	fmol C
**Pool C:N ratios**			
CN_{NMR}	C:N ratio of complex N-rich	5	

	compounds (a mixture of proteins, DNA, RNA, as in $C_{NMR,}$ C_{Enz} and F_{NC}*C_{BM})	
CN_{CMR}	C:N ratio of complex C-rich compounds (a mixture of microbial cell walls, lipids, starch as in C_{CMR} and F_{CC}*C_{BM})	150
CN_{PS}	C:N ratio of primary substrate (plant material)	Depending on scenario (5-80)
$CN_{MR\text{-}DOM}$	C:N ratio of the microbes internal pool of solubles (F_{DOM}*C_{BM})	15
**Initial number of microsites occupied by microbes (randomly distributed over 10.000 microsites). Each microbe starts with ½ of its max cell size**		
MO	Microsites occupied by microbes	1666

Parameter values are derived from a Bayesian calibration (Markov-Chain Monte Carlo simulation), based on literature values and empirical data obtained from a litter decomposition experiment in mesocosms (for details see supporting material in [1]). *Prior ranges for enzyme kinetics parameters (k_{cat} and k_m) were derived from the literature [6,7]: For achieving turnover number (k_{cat}) of enzymes, we divided published v_{max} values by an estimated enzyme concentration of 1/10 of the microbial biomass in decomposing litter. One microsite = 1000 µm^3. §Assuming leaf density: 500 µg/mm^3 [8]; water content: 60%, C content: 50% dry mass. The C:N ratio of bioavailable dissolved organic matter (CN_{DOM}) has an initial value of 8, it is however not a parameter but a variable which can change over the course of the model run. One model time-step=3 hours.

Supplementary Table 2: Overview over C and N transformations within one microsite in each time step.

Pool description	Pool C/N ratio	Enzyme needed for break-down	C or N part of the pool		C and N flow to/from the pool due to enzymatic breakdown	C and N flow due to microbial mortality and inactivation of enzymes	C and N flow due to microbial metabolism	C and N flow due to diffusion[3]
**Primary substrate (PS)**	CN_{PS} [given by scenario]	C_{enzPS}	C	C_{PS}	$-d_{\mathrm{C\_PS}}$	-		
			N	$C_{\mathrm{PS}}/CN_{\mathrm{PS}}$				
**C-rich complex compounds (CMR)**	CN_{CMR} [=150]	C_{enzCMR}	C	C_{CMR}	$-d_{\mathrm{C\_CMR}}$	$+C_{\mathrm{deathBM}}\ F_{\mathrm{CC}}$		
			N	$C_{\mathrm{CMR}}/CN_{\mathrm{CMR}}$		$+C_{\mathrm{deathBM}}\ F_{\mathrm{CC}}/CN_{\mathrm{CMR}}$		
**N-rich complex compounds (NMR)**	CN_{NMR} [=5]	C_{enzNMR}	C	C_{NMR}	$-d_{\mathrm{C\_NMR}}$	$+C_{\mathrm{deathBM}}\ F_{\mathrm{NC}}$ $+C_{\mathrm{enz}}\ k_{\mathrm{enz}}$		
			N	$C_{\mathrm{NMR}}/CN_{\mathrm{NMR}}$		$+C_{\mathrm{deathBM}}\ F_{\mathrm{NC}}/CN_{\mathrm{NMR}}$ $+C_{\mathrm{enzX\_}}\ k_{\mathrm{enz}}/CN_{\mathrm{NMR}}$		
**Bioavailable dissolved organic matter (DOM)**	CN_{DOM} [$= C_{\mathrm{DOM}}/N_{\mathrm{DOM}}$]		C	C_{DOM}	$+d_{\mathrm{C\_PS}}$ $+d_{\mathrm{C\_CMR}}$ $+d_{\mathrm{C\_NMR}}$	$+C_{\mathrm{deathBM}}\ F_{\mathrm{DOM}}$	$-C_{\mathrm{upt}}$	$-n\ \frac{C_{\mathrm{DOM}}/(1+frL)}{n+1}+\sum_{i=0}^{n}\frac{C_{\mathrm{DOM}_i}/(1+frL)}{n+1}$
			N	N_{DOM}	$+d_{\mathrm{C\_PS}}/CN_{\mathrm{PS}}$ $+d_{\mathrm{C\_CMR}}/CN_{\mathrm{CMR}}$ $+d_{\mathrm{C\_NMR}}/CN_{\mathrm{NMR}}$	$+C_{\mathrm{deathBM}}\ F_{\mathrm{DOM}}$ $/CN_{\mathrm{MB}}$	$-N_{\mathrm{DON\_upt}}$	$-n\ \frac{N_{\mathrm{DOM}}/(1+frL)}{n+1}+\sum_{i=0}^{n}\frac{N_{\mathrm{DOM}_i}/(1+frL)}{n+1}$
**Dissolved inorganic nitrogen (DIN)**	-		N	N_{IN}			$-N_{\mathrm{imm}}+N_{\mathrm{min}}$	$-n\ \frac{N_{\mathrm{in}}/(1+frL)}{n+1}+\sum_{i=0}^{n}\frac{N_{\mathrm{in}_i}/(1+frL)}{n+1}$
**Microbial biomass (BM)**	CN_{BM} [deter-mined by microbial functional group]		C	C_{BM}		$-C_{\mathrm{deathBM}}$ [2]	$+C_{\mathrm{upt}}$ $-C_{\mathrm{resp}}$ $-C_{\mathrm{enz\_prod}}$ [1]	
			N	$C_{\mathrm{BM}}/CN_{\mathrm{BM}}$		$-C_{\mathrm{deathBM}}/CN_{\mathrm{BM}}$	$+N_{\mathrm{DON\_upt}}$ $+N_{\mathrm{imm}}-N_{\mathrm{min}}$	
**Extra-cellular Enzymes**	CN_{NMR}[=5]		C	C_{enzX} [5]		$-C_{\mathrm{enzX}}\ k_{\mathrm{enz}}$ [4,6]	$+C_{\mathrm{enz\_prod}}\ E_{\mathrm{fX}}$	
			N	$C_{\mathrm{enzX}}/CN_{\mathrm{NMR}}$		$-C_{\mathrm{enzX}}k_{\mathrm{enz}}/CN_{\mathrm{NMR}}$	$+C_{\mathrm{enz\_prod}}E_{\mathrm{fX}}/CN_{\mathrm{NMR}}$	

All units (except C/N ratios and fractions such as E_{fx}) are in mol C and mol N, respectively

[1]C_{resp} includes Respiration for Maintenance, Enzyme Production and Growth, as well as Overflow respiration.

[2]If the microbe present at this microsite dies in this timestep, 100% of C_{BM} is transferred to $C_{deathBM}$, otherwise $C_{deathBM}$ = 0.

[3] Diffusion is modelled as a brownian motion of DOM/DIN particles on the grid. For a description of the diffusion algorithm see Supplementary Note 1. i is one out of n neighbouring microsites (n=8). frL is the fraction of diffusing elements that is lost from the grid by leaching (Table 1). The amount of DOM (or DIN) lost by leaching is consequently (per microsite): $n\frac{C_{\mathrm{DOM}}\,frL/(1+frL)}{n+1}$

[4]k_{enz} = First order rate constant for inactivation of enzymes. $1/k_{enz}$ is the mean life-time of enzymes. Upon inactivation, enzymes are transferred to the C_{NMR} pool, where they will be subject to degradation by extracellular enzymes themselves.

[5] C_{enzX} can be C_{enz_PS}, C_{enz_CMR} and C_{enz_NMR}

[6] E_{fx} can be E_{fPM}, E_{fCR} or E_{FNR}

Supplementary Methods

Calculation of C and N transformations in the model

The following calculations are carried out for each microsite in each time step. Microsites are processed in a random order. Microbial dispersal as well as diffusion of bioavailable dissolved organic matter (DOM) and dissolved inorganic nitrogen (DIN) are modelled at the grid level. Units are, unless otherwise indicated, mol C for variables whose names start with a "C" and mol N for those starting with a "N" (both directly followed by a subscript, f.e. $C_{\text{resp\_maint}}$ or N_{imm}) .

Enzymatic breakdown of complex substrates

Extracellular enzymes break down complex substrate each time-step in each microsite according to Michaelis-Menten kinetics [9,7] :

$$d_{\text{c}} = k_{\text{cat\_x}}\, C_{\text{Enz\_x}}\, \frac{C_{\text{X}}}{k_{\text{m\_x}} + C_{\text{X}}} \qquad \text{[mol C]}$$

$d_{\text{c\_x}}$ Amount of C released by enzyme-catalysed breakdown of a complex substrate in one microsite (in mol C transferred from the complex substrate to the bioavailable DOM pool per time step). X can be PS (Primary substrate = plant material), CMR (C-rich microbial remains) or NMR (N-rich microbial remains).

$k_{\text{cat\_x}}$ Catalytic constant: number of enzymatic reactions catalyzed per time step per enzyme (mol substrate-C decomposed per mol enzyme-C). Can be $k_{\text{cat\_PM}}$, $k_{\text{cat\_CMR}}$, $k_{\text{cat\_MNR}}$

C_{X} Amount of complex substrate in this microsite, can be C_{PS} , C_{CMR}, C_{NMR} (mol C)

$k_{\text{m\_x}}$ Half saturation constant for substrate (mol C). Can be $k_{\text{m\_PS}}$, $k_{\text{m\_CMR}}$, $k_{\text{m\_NMR}}$.

$C_{\text{Enz\_x}}$ Amount of enzymes present in this microsite (mol C). Can be $C_{\text{Enz\_PS}}$, $C_{\text{Enz\_CMR}}$, $C_{\text{Enz\_NMR}}$.

C and N released from enzyme-catalysed breakdown of complex compounds are added to the pool of bioavailable dissolved organic matter (DOM) of the respective microsite (see below).

Microbial Uptake of C and N

Microbes take up C and N from the labile pools (DOM and DIN) in their microsite subject to their physiological limit *and* to the amount of DOM and DIN present in their microsite. First, potential maximum (physiological) C uptake of a microbe (U_{pot}) is calculated based on its biomass and surface:volume ratio:

Cell volume is derived from biomass-C,

$$V_{\mathrm{cell}} = C_{\mathrm{BM}}\, m_{\mathrm{C}}\, 10 \qquad [\mathrm{cm}^3]$$

Assuming the density of a microbial cell to be 1000 fg fresh weight μm^{-3} (1g cm^{-3}); with C accounting for 10% of fresh weight (assuming 80% water content and C accounts for 50% of dry weight). [5]. C_{BM} is biomass-C of the microbial cell in the model (in mol), m_C is the molar weight of C (12 g mol^{-1}), and V_{cell} is the volume of a microbial cell in cm^3.

Surface/volume ratio is calculated, assuming a spheric shape:

$$R_{\mathrm{sv}} = 3/\sqrt[3]{\frac{3V_{\mathrm{cell}}}{4\pi}}$$

We assume that the smaller a microbe is, the more substrate it can take up in relation to its biomass (because of larger surface:volume ratios). To link uptake to surface area, the basic maximum uptake rate is multiplied with the surface to volume ratio:

$$U_{\mathrm{adj}} = U_{\mathrm{max}}\, R_{\mathrm{sv}}$$

Where U_{adj} is the adjusted max C uptake rate as a fraction of biomass by this microbe in one time step. U_{max} is the basic maximum uptake rate, as defined in the general parameter settings (Supplementary Table 1).

$$U_{\mathrm{pot}} = U_{\mathrm{adj}}\, C_{\mathrm{BM}} \qquad [\mathrm{mol\ C}]$$

U_{pot} is the potential maximum C uptake (in mol C) by this microbe in this time step (given sufficient substrate availability).

C is taken up subject to this individual maximum uptake rate *and* to its availability at the microsite (up to a maximum of 95% of the available DOM in the microsite):

$$If\ (U_{\mathrm{pot}} > C_{\mathrm{DOM}} \times 0.95)\ then\ C_{\mathrm{upt}} = C_{\mathrm{DOM}} \times 0.95\ \ else\ C_{\mathrm{upt}} = U_{\mathrm{pot}}$$

Where C_{upt} is the amount of C effectively taken up by the microbe this time-step.

N is taken up from the microsite's DOM pool according to the current pool C:N ratio (CN_{DOM}). In addition, up to 95% of the N in the DIN pool of the microsite can be taken up. This is modelled as a temporary uptake of N_{in}*0.95, as every N in excess will be released again to the DIN pool at the end of the time-step.

$$N_{\mathrm{DON\_upt}} = C_{\mathrm{upt}}/CN_{\mathrm{DOM}}$$

$$N_{\text{imm}} = N_{\text{in}} \times 0.95$$

$$N_{\text{upt}} = N_{DON_{\text{upt}}} + N_{\text{imm}}$$

Where N_{DON_upt} is N uptake from the DOM pool and N_{imm} is total (gross) N immobilisation (i.e. uptake from the inorganic N pool) at this microsite.

These uptakes reduce the respective DOM and DIN pools in the microsite (see below).

Maintenance respiration

Maintenance respiration needs to be met first, before investing resources into enzyme production and growth.

A. If there are enough resources from C uptake to meet maintenance respiration (**C_{upt} >= C_{BM}*R_{maint}**), they are used for respiration:

$$C_{\text{resp\_maint}} = C_{\text{BM}} R_{\text{maint}}$$

$$C_{\text{upt\_remM}} = C_{\text{upt}} - C_{\text{resp\_maint}}$$

$$N_{\text{upt\_remM}} = N_{\text{upt}}$$

Where C_{resp_maint} is the amount of C respired for maintenance of biomass (in mol C), and C_{upt_remM} and N_{upt_remM} are the amounts of C and N still available for enzyme production and growth.

B. Otherwise **(if C_{upt} < C_{BM}*R_{maint})**, the missing C amount to carry out maintenance respiration is deducted from the microbe's biomass:

In this case, if microbial biomass is large enough to cover the missing C for maintenance respiration (If C_{BM} >= C_{BM}*R_{maint} – C_{upt}):

$$C_{\text{resp\_maint}} = C_{\text{BM}} R_{\text{maint}}$$

$$C_{\text{BM\_loss}} = C_{\text{BM}} R_{\text{maint}} - C_{\text{upt}}$$

If not (Else), total microbial biomass is lost (leading to the death of the microbe):

$$C_{\text{resp\_maint}} = C_{\text{upt}} + C_{\text{BM}}$$

$$C_{\text{BM\_loss}} = C_{\text{BM}}$$

In both cases of B, no C is left from uptake to be invested into growth or enzyme production (C_{upt_rem} = 0). By respiring biomass C for maintenance purposes, associated biomass N resources are liberated (because the microbe's C:N ratio is kept constant). This N adds to $N_{upt\_rem,}$ which will be excreted to N_{in} in the end of the timestep.

$$C_{\text{upt\_remM}} = 0$$

$$N_{\text{upt\_remM}} = N_{\text{upt}} + C_{\text{BM\_loss}} / M_{\text{CN}}$$

$C_{\text{upt\_remM}}$ and $N_{\text{upt\_remM}}$ are the amounts of C and N from uptake still available for enzyme production and growth (after deduction of maintenance respiration).

Microbial enzyme production

If there are still resources available **($C_{\text{upt\_rem}} > 0$)** after deduction of maintenance respiration, they are first used for enzyme production (up to a functional-group specific fraction) and the rest of it is used for growth. Enzyme production thus reduces resources available for growth.

The amount of C a microbe invests into extracellular enzyme production is defined by a functional group-specific fraction (E_{fr}) of the C remaining from uptake after deduction of maintenance respiration ($C_{\text{upt\_rem}}$). The C and N needed to produce enzymes is temporarily calculated as:

$$C_{\text{enz\_prod}} = C_{\text{upt\_remM}} \, E_{\text{fr}}$$

$$C_{\text{costs\_enz}} = C_{\text{enz\_prod}}/(1 - R_{\text{ge}})$$

$$N_{\text{enz\_prod}} = C_{\text{enz\_prod}} / CN_{\text{NMR}}$$

Where $C_{\text{enz\_prod}}$ is the amount of C in extracellular enzymes that are to be produced in that time step, and $C_{\text{costs\_enz}}$ is the total C investment needed for enzyme production (including respiration associated with enzyme production). $N_{\text{enz\_prod}}$ is the N needed for enzyme production. CN_{NMR} is the C:N ratio of N-rich complex substrates (which also encompasses enzymes) in the model ($CN_{\text{NMR}} = 5$).

If both, ($C_{\text{costs\_enz}} <= C_{\text{upt\_remM}}$) and ($N_{\text{enz\_prod}} <= N_{\text{upt}}$), enzymes are produced as outlined above.

If enzyme production is N limited ($N_{\text{enz\_prod}} > N_{\text{upt}}$), but not C limited ($C_{\text{costs\_enz}} <= C_{\text{upt\_remM}}$) enzymes are produced up to the N limit (replaces temporary calculations above):

$$N_{\text{enz\_prod}} = N_{\text{upt}}$$

$$C_{\text{enz\_prod}} = N_{\text{enz\_prod}} \, CN_{\text{NMR}}$$

$$C_{\text{costs\_enz}} = C_{\text{enz\_prod}}/(1 - R_{\text{ge}})$$

If enzyme production is C limited ($C_{\text{costs\_enz}} > C_{\text{upt\_remM}}$), but not N limited ($N_{\text{enz\_prod}} <= N_{\text{upt}}$) enzymes are produced up to the C limit (replaces temporary calculations above):

$$C_{\text{costs\_enz}} = C_{\text{upt\_remM}}$$

$$C_{\text{enz\_prod}} = C_{\text{costs\_enz}}(1 - R_{\text{ge}})$$

$$N_{\text{enz\_prod}} = C_{\text{costs\_enz}}(1 - R_{\text{ge}})/CN_{\text{NMR}}$$

If enzyme production is both C and N limited, Enzymes are produced up to the N limit (see above). Negative C is then deducted from biomass.

In case of insufficient resources, a constitutive minimum of 1/10 of the anticipated enzyme production is carried out even at the expense of biomass (Details not shown, analogue to maintenance respiration).

$$C_{\text{enz\_const}} = S_{\max}(U_{\text{adj}} - R_{\text{maint}})\frac{E_{\text{fr}}}{10}$$

where $S_{\max}$ is the maximum cell size of the microbe (in mol) and $C_{\text{enz\_const}}$ the amount of C needed for constitutive enzyme production.

Respiration associated with Enzyme production is calculated as:

$$C_{\text{resp\_enz}} = C_{\text{costs\_enz}} - C_{\text{enz\_prod}}$$

Total enzyme production of a microbe is distributed among the three different enzyme classes present in the microsite according to functional-group specific production ratios (see below).

Enzyme lifetime

Active extracellular enzymes are assumed to become inactivated after some time. The time an enzyme keeps active after its production is regulated by a first-order rate constant (k_{enz}), whose inverse ($1/k_{\text{enz}}$) is thereby the mean lifetime of extracellular enzymes in the model.

Overall change of the enzyme pools in a microsite over time:

$$C_{\text{enzPM}_{t+1}} = C_{\text{enzPS}} + C_{\text{enz\_prod}}E_{\text{fPS}} - C_{\text{enzPS}}\,k_{\text{enz}}$$

$$C_{\text{enzCMR}_{t+1}} = C_{\text{enzCMR}} + C_{\text{enz\_prod}}E_{\text{fCMR}} - C_{\text{enzCMR}}\,k_{\text{enz}}$$

$$C_{\text{enzNMR}_{t+1}} = C_{\text{enzNMR}} + C_{\text{enz\_prod}}E_{\text{fNMR}} - C_{\text{enzNMR}}\,k_{\text{enz}}$$

Where $C_{\text{enz\_PM}}$, $C_{\text{enz\_CMR}}$ and $C_{\text{enz\_NMR}}$ are the respective enzyme pools for enzymes degrading plant material, C-rich and N-rich microbial remains in this microsite. E_{fPM}, E_{fCR} and E_{fNR} are the fractions of total enzyme production invested into each of this enzyme classes by this microbial functional group (Table 1) and t is the current time-step.

Inactivated Enzymes are transferred to the pool of N-rich complex compounds (C_{NMR}) in the microsite where they are subject to decay by extracellular enzymes themselves (see summary equation for C_{NMR} below).

C and N remaining from uptake after deduction of maintenance respiration and enzyme production are calculated:

$$C_{\text{upt\_remE}} = C_{\text{upt\_remM}} - C_{\text{costs\_enz}}$$

$N_{\text{upt\_remE}} = N_{\text{upt\_remM}} - N_{\text{enz\_prod}}$

Microbial Growth

The possible microbial growth is first assessed from the rest of C available after deduction of Enzyme production:

$C_{\text{growthP}} = C_{\text{upt\_remE}} (1 - R_{\text{ge}})$

If possible microbial growth turns out to be C limited **($(C_{\text{growthP}}/N_{\text{upt\_remE}}) <= M_{\text{CN}}$),** all C from uptake is used for growth while part of N that had been taken up remains in excess ($N_{\text{upt\_remG}}$) :

$$C_{\text{growth}} = C_{\text{upt\_remE}} (1 - R_{\text{ge}})$$

$$C_{\text{resp\_growth}} = C_{\text{upt\_remE}} R_{\text{ge}}$$

$$C_{\text{upt\_remG}} = 0$$

$$N_{\text{upt\_remG}} = N_{\text{upt\_remE}} - C_{\text{growth}}/M_{\text{CN}}$$

Otherwise, if microbial growth is N limited **($(C_{\text{growthP}}/N_{\text{upt\_remE}}) > M_{\text{CN}}$)**, biomass grows to the N limit and C remains in excess ($C_{\text{upt\_rem}}$):

$$C_{\text{growth}} = N_{\text{upt\_remE}} M_{\text{CN}}$$

$$N_{\text{upt\_remG}} = 0$$

$$C_{\text{resp\_growth}} = \left(\frac{C_{\text{growth}}}{1 - R_{\text{ge}}}\right) R_{\text{ge}}$$

$$C_{\text{upt\_remG}} = C_{\text{upt\_remE}} - C_{\text{growth}}/(1 - R_{\text{ge}})$$

Change in microbial biomass over time is calculated by adding growth (if it has happened) and subtracting possible losses (i.e. by starvation, when maintenance respiration and/or constitutive enzyme production has to have been deducted from biomass)

$C_{\text{BM}_{t+1}} = C_{\text{BM}} + C_{\text{growth}} - C_{\text{BM\_Loss}}$

Stoichiometric waste metabolism:

<u>Mineralisation:</u> N remaining from uptake is released to the inorganic N pool (N_{in}) of the microsite (gross N mineralization, N_{min})

$N_{\text{min}} = N_{\text{upt\_remG}}$

The difference between initial N uptake from the inorganic N pool (N_{imm}) and total release of excess N (N_{min}) depicts the net exchange between the microbe and the inorganic N pool of this microsite in this timestep (net mineralization, N_{netmin}) :

$$N_{\text{netmin}} = N_{\text{min}} - N_{\text{imm}}$$

<u>Overflow respiration:</u> C remaining from uptake is respired as overflow respiration:

$$C_{\text{resp\_overflow}} = C_{\text{upt\_remG}}$$

Total respiration:

Total respiration in this microsite and time step finally adds up to:

$$C_{\text{resp}} = C_{\text{resp\_maint}} + C_{\text{resp\_enz}} + C_{\text{resp\_growth}} + C_{\text{resp\_overflow}}$$

Recycling of microbial biomass

There are two ways to die for a microbe in the model. One is by starvation (which happens, if biomass falls below a minimum limit, i.e. $C_{\text{BM}} < S_{\text{min}}$). The second way to die is due to 'catastrophic death' (reflecting predation or abrupt changes of environmental conditions). The latter is implemented as a functional group-specific probability of each individual to die in each time step (stochastic mortality rate *m*).

Stochastic mortality rates (*m*) are linked to the maximum cell size of the functional group (S_{max}). We assume that species with larger cells invest more in structural and/or defensive cell compounds, which makes them more resistant against catastrophic death. Smaller cells have thus a higher chance to die than larger cells. We implemented this assumption by relating mortality rate inversely to S_{max} (Table 1).

$$m = \frac{1}{S_{\text{max}}} m_{\text{f}}$$

Where m_{f} is the factor relating mortality rate to the inverse of maximum biomass (Table 1), S_{max} is the maximum cell size (in fmol C) and m is the probability of a microbe to die in one time step by catastrophic death.

Each time step each a floating-point decimal between 0 and 1 is generated by a random number generator for each individual microbe in the model (*r*). If, for any microbe, this number happens to be lower than the microbial mortality rate ($r<m$), OR microbial biomass has fallen below the lower limit ($C_{\text{BM}} < S_{\text{min}}$) the microbe dies:

$$C_{\text{deathBM}} = C_{\text{BM}}$$

$$C_{\text{BM}} = 0$$

Otherwise, not:

$$C_{\text{deathBM}} = 0$$

Upon cell death, the biomass C and N of the microbe is distributed among different substrate pools within the local microsite according to their functional-group dependent biomass composition (determined by F_{CC},F_{NC} and F_{DOM}, Table 1). These substrate pools change consequently with time like this:

$$C_{\text{CMR}_{t+1}} = C_{\text{CMR}} + C_{\text{deathBM}} F_{\text{CC}}$$

$$C_{\text{NMR}_{t+1}} = C_{\text{NMR}} + C_{\text{enz\_PS}}\ k_{\text{enz}} + C_{\text{enz\_NMR}}\ k_{\text{enz}} + C_{\text{enz\_CMR}}\ k_{\text{enz}} + C_{\text{deathBM}}\ F_{\text{NC}}$$

$$C_{\text{DOM}_{t+1}} = C_{\text{DOM}} + \cdots + C_{\text{deathBM}} F_{\text{DOM}}$$

Note that the C_{NMR} pool additional receives input from inactivated extracellular enzymes.

Each of the three biomass components, which make up a microbial cell in the model, has a distinct C:N ratio (Table 1). Every microbial functional group is defined to have a certain relative composition of the three biomass components (as determined by F_{DOM}, F_{CC}, F_{NC}), which in turn determines overall microbial cell C:N ratio (Supporting information Table 1).

The N component of the C_{CMR} and C_{NMR} pools are not explicitely modelled, as these pools have a fixed C:N ratio, which is the same as the C:N ratio of their inputs (CN_{CMR}= 150, CN_{NMR}= 5). The N_{DOM} pool, however, has a variable C:N ratio due to various inputs, and is thus explicitely modelled:

$$N_{\text{DOM}_{t+1}} = N_{\text{DOM}} + \cdots + C_{\text{deathBM}} F_{\text{DOM}} / CN_{\text{MR-DOM}}$$

Where $CN_{MR\text{-}DOM}$ is the C:N ratio of the internal pool of solubles in microbes (=15).

For the full form of both, C_{DOM} and N_{DOM} equations, see below.

Microbial dispersal

Mortality (stochastic or by starvation) creates empty microsites, which will be occupied by the most successful microbes in their surrounding: Microbial cells divide and colonize a neighbouring microsite, if their biomass exceeds their functional group-specific maximum level (S_{max}). If all neighboring microsites are occupied, microbes can ‘invade’ an occupied microsite with a probability of 0.01 (leading to the death of the owner). Microbes are not ‘mobile’ in the model. The only movement of microbes on the grid is due to dispersal to neighboring microsites in the course of reproduction.

Diffusion

Elements of C_{DOM}, N_{DOM} and N_{IN} are mixing via diffusion across the grid. For details, and how this is connected to empirical diffusion coefficients see Supplementary material in [1]. Briefly, we assume that each microsite equilibrates C_{DOM}, N_{DOM} and N_{IN} with its nearest neighbouring microsites in one time step. For that means, each microsite loses 1/(n+1) of its C_{DOM}, N_{DOM}

and N_{IN} pools to each of its adjacent neighbouring microsites, with n+1 being the number of adjacent neighbours(n=8) plus the source microsite itself. A small part of this diffusing elements ($C_{DOM}*frL/(1+frL)$) is lost by leaching during the transition. Diffusion is carried out in a random order across the grid with temporary results being copied into a temporary copy grid in order to avoid multiple counting of diffusing elements.

$$C_{\mathrm{DOM}_{t+1}} = C_{\mathrm{DOM}} + \cdots - n\,\frac{C_{\mathrm{DOM}}/(1+frL)}{n+1} + \sum_{i=0}^{n} \frac{C_{\mathrm{DOM}_i}/(1+frL)}{n+1}$$

Where *frL* is the fraction of diffusing elements lost by leaching, n is the number of neighbouring cells within which element concentration is equilibrating within one time step.

$$C_{\mathrm{Leach}} = n\frac{C_{\mathrm{DOM}}\,frL/(1+frL)}{n+1}$$

C_{Leach} is the amount of C_{DOM} lost by leaching from this microsite (analogue for N_{DOM} and N_{IN}).

Overall pool changes within one microsite and effect on DOM C:N ratio

In sum, the pool sizes of C_{DOM} , N_{DOM} and N_{in} will alter in any microsite each time step as follows:

$$C_{\mathrm{DOM}_{t+1}} = C_{\mathrm{DOM}} + C_{\mathrm{deathBM}}F_{\mathrm{DOM}} + d_{\mathrm{c\_PS}} + d_{\mathrm{C\_CMR}} + d_{\mathrm{C\_NMR}} - C_{\mathrm{upt}} - n\,\frac{C_{\mathrm{DOM}}/(1+frL)}{n+1} + \sum_{i=0}^{n} \frac{C_{\mathrm{DOM}_i}/(1+frL)}{n+1}$$

$$N_{\mathrm{DOM}_{t+1}} = N_{\mathrm{DOM}} + C_{\mathrm{deathBM}}F_{\mathrm{DOM}}/CN_{\mathrm{MR-DOM}} + d_{\mathrm{c_{PS}}}/CN_{\mathrm{PS}} + d_{\mathrm{C_{CMR}}}/CN_{\mathrm{CMR}} + d_{\mathrm{C_{NMR}}}/CN_{\mathrm{NMR}} - N_{\mathrm{DON\_upt}} - n\,\frac{N_{\mathrm{DOM}}/(1+frL)}{n+1} + \sum_{i=0}^{n} \frac{N_{\mathrm{DOM}_i}/(1+frL)}{n+1}$$

$$N_{\mathrm{in}_{t+1}} = N_{\mathrm{in}} - N_{\mathrm{imm}} + N_{\mathrm{min}} - n\,\frac{N_{\mathrm{in}}/(1+frL)}{n+1} + \sum_{i=0}^{n} \frac{N_{\mathrm{in}_i}/(1+frL)}{n+1}$$

As DOM receives inputs from sources with distinct C:N ratios (f.e. dead microbial biomass DOM, DOM liberated from enzymatic degradation of the complex substrate pools C_{CMR}, C_{NMR} and C_{PS}, diffusing DOM) whose relative fractions vary across time and space (subject to

enzyme activity, substrate availability, microbial mortality and local interactions), C:N ratio of the DOM pool is variable and can be calculated as:

$$CN_{\mathrm{DOM}} = C_{\mathrm{DOM}}/N_{\mathrm{DOM}}$$

Aggregated model output

For calculating aggregated model output, pool sizes of all microsites are summed up for the whole grid and related to the size of the grid (Grid = 10.000 microsites of each 1000 μm^3=0.01 mm^3). Fluxes are additional adjusted to time (1 model time step = 3 hours). Initial primary substrate C on the grid (= 8333 fmol plant material C / microsite) was chosen by assuming a leaf density of 500 $\mu g/mm^3$ [8] a water content of 60%, and C content of 50% dry mass.

Supplementary References

1. Kaiser, C., Franklin, O., Dieckmann, U. & Richter, A. Microbial community dynamics alleviate stoichiometric constraints during litter decay. *Ecol. Lett.* **17,** 680–690 (2014).

2. Kirchman, D. L. *Processes in microbial ecology*. 24 (Oxford University Press, 2012).

3. Bryan, A. K., Goranov, A., Amon, A. & Manalis, S. R. Measurement of mass, density, and volume during the cell cycle of yeast. *Proc. Natl. Acad. Sci. U. S. A.* **107,** 999–1004 (2010).

4. Rutz, B. A. & Kieft, T. L. Phylogenetic characterization of dwarf archaea and bacteria from a semiarid soil. *Soil Biol. Biochem.* **36,** 825–833 (2004).

5. Romanova, N. D. & Sazhin, A. F. Relationships between the cell volume and the carbon content of bacteria. *Oceanology* **50,** 522–530 (2010).

6. German, D. P., Marcelo, K. R. B., Stone, M. M. & Allison, S. D. The Michaelis-Menten kinetics of soil extracellular enzymes in response to temperature: a cross-latitudinal study. *Glob. Chang. Biol.* **18,** 1468–1479 (2012).

7. Wang, G., Post, W. M., Mayes, M. A., Frerichs, J. T. & Sindhu, J. Parameter estimation for models of ligninolytic and cellulolytic enzyme kinetics. *Soil Biol. Biochem.* **48,** 28–38 (2012).

8. Witkowski, E. T. F. & Lamont, B. B. Leaf specific mass confounds leaf density and thickness. *Oecologia* 486–493 (1991).

9. Allison, S. D., Wallenstein, M. D. & Bradford, M. A. Soil-carbon response to warming dependent on microbial physiology. *Nat. Geosci.* **3,** 336–340 (2010).
